# Trends in energy and nutrient content of menu items served by large UK chain restaurants from 2018 to 2020: an observational study

**DOI:** 10.1136/bmjopen-2021-054804

**Published:** 2021-12-30

**Authors:** Yuru Huang, Dolly R Z Theis, Thomas Burgoine, Jean Adams

**Affiliations:** MRC Epidemiology Unit, Cambridge University, Cambridge, UK

**Keywords:** epidemiology, health policy, public health, nutrition & dietetics

## Abstract

**Objective:**

The objective of this study was to evaluate the change in energy and nutrient content of menu items sold in large UK chain restaurants (eg, fast food, full service) from 2018 to 2020.

**Design:**

Observational study.

**Setting:**

Energy and nutritional information of menu items served by 29 large UK chain restaurants that consistently provided this information online in all three years. Data were collected in 2018 (March–April), 2019 (April) and 2020 (October–November) from restaurant websites.

**Primary and secondary outcome measures:**

The per-item energy and nutrient (saturated fat, sugar and salt) changes in all items available on menus (‘all menu items’) and recurring items that were consistently available on menus in all three years (‘core menu items’), overall and in 12 different food categories.

**Results:**

Our study included 7770, 9213 and 6928 menu items served by 29 large UK chain restaurants in 2018, 2019 and 2020, respectively. Our results showed that sugar content declined from 2018 to 2020 among all menu items (per-item: −0.43 g/year, 95% CI −0.66 to –0.21). This reduction in sugar was evident in beverages, sandwiches and desserts. Among core menu items (N=1855), sugar content reduced significantly from 2018 to 2020 (per-item: −0.31 g/year, 95% CI −0.45 to –0.17), especially in beverages. Energy, salt and saturated fat content in menu items remained constant overall, in both all menu items and core menu items. Fewer food categories had significant changes in energy, sugar, salt and saturated fat content among core menu items than among all menu items.

**Conclusions:**

From 2018 to 2020, sugar content declined in restaurant menu items, which may reflect a response to the sugar reduction strategy and the effects of the soft drinks industry levy. In contrast, there was little change in other nutrients. Future policies addressing the overall nutritional quality of restaurant foods, rather than single nutrients, may help the restaurant sector move towards offering healthier foods.

**Strengths and limitations of this study**

- This is the first study to evaluate longitudinal trends in energy and nutrient content of menu items served by large chain restaurants in the UK.
- The study used data on the same 29 large chain restaurants across three years to enable comparison.
- Core menu items present in all three years were identified, allowing us to examine whether menu item level reformulation had occurred during this time period.
- Only items presented on online menus were included, which may differ from items available in-store.
- This is an analysis of a three-year menu change among large chain restaurants and does not include independent retailers or other smaller chains.

## Background

Globally, foods prepared out-of-home by restaurants, cafes, takeaways and similar outlets tend to be energy-dense and nutrient-poor.^1–5^ Internationally, 94% of meals served by full service restaurants and 72% served by fast food outlets exceeded the government recommendation of no more than 600 calories for lunch or dinner.^5^ In the UK, comparable figures were 96% and 70%.^3^ The frequency of eating food prepared out-of-home is also increasing. In the UK, one study estimated that over a quarter of adults consumed food prepared out-of-home once a week or more.^6^ Similar eating patterns were also reported in Western countries such as the US and other European countries.^7 8^ Eating out more frequently is associated with increased energy intake, lower diet quality and elevated body weight, which in turn are associated with a number of chronic non-communicable diseases.^9–13^

Small changes in the energy and nutrient content of large chain restaurant menu items may, therefore, impact population dietary intake, and ultimately, health. Recent studies suggest that chain restaurants in Western countries have made varying levels of progress towards improving the energy and nutrient content of menu items.^14–17^ In the USA, the energy and nutrient content of newly introduced items declined between 2012 and 2018.^14^ In New Zealand, energy and sodium per serve of fast foods increased substantially from 2012 to 2016.^16^ In Ontario, Canada, the mean energy content of menu items served by chain restaurants increased between 2010 and 2017.^17^

It is possible that changes in the energy and nutrient content of restaurant menu items is influenced by local policy context.^14^ The UK government has implemented several recent policies to encourage healthier practices in the out-of-home food sector.^18^ However, these have largely been voluntary programmes, whereby voluntary reduction targets are set for manufacturers and retailers, including out-of-home food businesses.^19–22^ The UK’s national salt reduction programme started in 2003, and guidelines for sugar and calorie reduction were introduced in 2017 and 2020, respectively.^19–22^ Although the progress of salt and sugar reduction programmes has been assessed, progress reports were based on limited out-of-home data and specific food categories, rather than whole menus.^23 24^ Mandatory calorie labelling for out-of-home food businesses was proposed by the UK government in 2018 and again in 2020, and is now due to come into force in April 2022.^25–29^ In the absence of this mandatory policy, evaluating whether and how the nutritional quality of restaurant foods has changed from 2018 to 2020 will help assess the impact of voluntary programmes and set a baseline for future independent and government evaluations of the mandatory calorie labelling policy.

In this study, we determined whether and how the energy and nutrient content of menu items sold in large chain restaurants in the UK, that consistently provided this information on their websites, changed over time from 2018 to 2020. We specifically examined trends in energy, sugar, saturated fat and salt content of items. As any overall trends might be due to changes in the nutrient profile of new menu items or the reformulation of existing menu items, we also examined changes in the energy and nutrient content of items available in all three years (hereinafter referred to as ‘core menu items’). Finally, we explored whether these trends varied by broad food categories.

## Methods

We collected data on the energy and nutrient content of foods served by large UK restaurant chains from their websites, annually from 2018 to 2020. We used this data to conduct longitudinal analyses of change in energy and nutrient content of all menu items, as well as core menu items.

### Restaurant inclusion criteria

**Figure 1:**
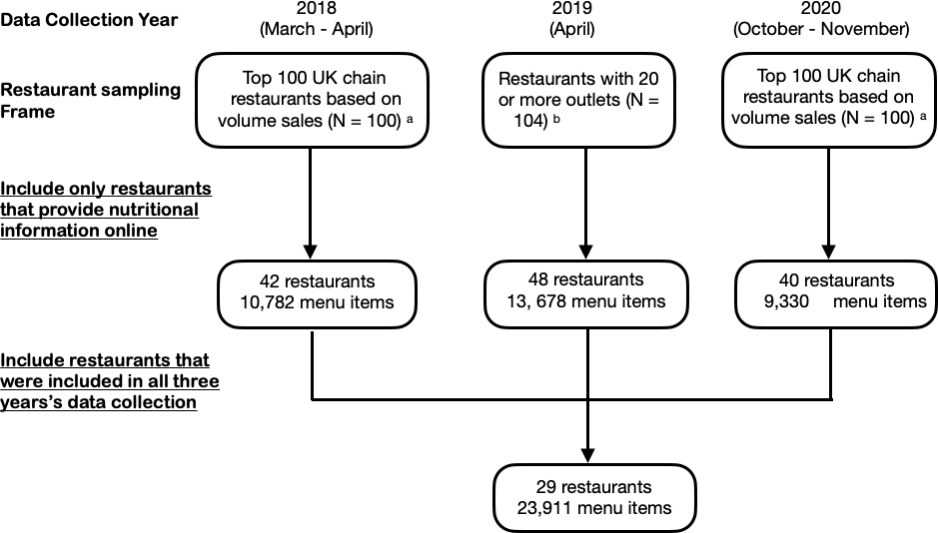
Restaurant inclusion criteria. ^a^ Based on technomic’s market repoer in 2013. ^b^ Based on Robinson et al’s list of restauants with 20 or more outlets in 2018.

Large chain restaurants were defined in 2018 and 2020 as those belonging to the top 100 businesses ranked by their total UK foodservice sales (based on a market research report in 2013), and in 2019 as those belonging to a chain with 20 or more outlets nationwide (based on a restaurant list derived in 2018).^30 31^ Energy and nutritional information of menu items served by these chain restaurants were collected in March–April 2018, April 2019 and October–November 2020. Large chain restaurants were included in the study if they provided nutritional information online for their menu items in all three years (figure 1). A total of 29 restaurant chains met the inclusion criteria (online supplemental materials, table S1).

10.1136/bmjopen-2021-054804.supp1 Supplementary data

### Menu item inclusion criteria

Information on menu items was collected for each restaurant as it appeared on websites. All food and drink items available at the restaurant for immediate consumption were included. Prepackaged items intended to be heated by customers for consumption at home (eg, packaged soups) were excluded. Menu items of different sizes (eg, individual, large pizza), those that were customisable (eg, milk choices for coffee), and those for consumption on and off the premises listed separately, were included as separate items to account for possible variations in energy and nutrient content.

### Data collection

Energy and nutritional information was collected from restaurants’ official websites. Details of the data collection methodology can be found elsewhere.^32^ Briefly, in 2018, the restaurant name, the names of individual menu items, their serving size and unit (eg, grams, ounces), and their total energy and nutrient content were transcribed from restaurant official websites into an excel spreadsheet. Menu item variations (eg, latte with oat/coconut drink) were recorded as separate entries. Components of a meal deal (or ‘combo meal’), eg, burger, fries and a beverage, were recorded as individual entries. Data on websites were converted to Excel tables using extraction tool ‘import.io’ in 2019.^33^ In 2020, we developed web crawlers using Python package Scrapy V.2.4, to automate part of the data collection process.^34^ Data from PDF tables were extracted using Python packages Tabula or Camelot.^35 36^ Alongside energy content in calories (kcal) and kilojoules (kJ), information on the following nutrients was extracted where available in all years: fat (g), saturated fat (g), carbohydrates (g), sugar (g), fibre (g), protein (g) and salt (g).

To minimise error arising as a result of inaccuracies presented on restaurant menus, we applied the following data cleaning rules:

1. For menu items that provided serving weight information online, the quantity of individual nutrients should not exceed the total serving weight. We set the gram weight or the nutrient value to missing if the quantity of a specific nutrient exceeded the total gram weight (N=54). If the nutrient value was not in the reasonable range (top or lower 5%), then the nutrient value was set to missing, otherwise the serving weight was set to missing. For example, the UK Classic Blueberry Muffin from Starbucks UK was listed as having 160 g of salt and a serving size of 116 g. As such, the salt value was set to missing.
2. Macronutrients and serving weight (g): For menu items that provided gram weight information online, the sum of all carbohydrates (g), protein (g) and fat (g) should not exceed the total gram weight. We set the gram weight to missing if the sum of all nutrient quantity exceeded the total gram weight (N=32). For example, the plain roast potatoes (40 g) from Toby Carvery were listed as containing 4 g of fat, 49 g of carbohydrates, and 6 g of protein. As such, the serving size was set to missing.
3. Duplicate items: We defined duplicate menu items as those with the same item name, nutritional information, restaurant and year. For example, chips (French fries) may appear on the same menu multiple times as standalone dishes, with the same nutritional values. A total of 491 duplicated menu items were deleted.

We standardised portion sizes for pizza items, if the nutritional information was presented by slice. For pizzas described in menus as ‘medium’, ‘large’, ‘family sized’ or ‘for sharing’, we calculated energy and nutrient content based on three slices of pizza. For pizzas described as ‘small’ or ‘for individual consumption’, the energy and nutrient content were calculated based on the whole pizza. This is consistent with how Domino’s pizza presented their nutritional information online.

### Menu item-level and restaurant-level characteristics

Menu items were deemed children’s menu items if the menu section, menu item name, or menu description contained ‘junior’, ‘kid’, ‘children’ or ‘child’. Menu items were ‘sharable’ if the menu section, menu item name or menu description contained ‘share’, ‘sharing’ or ‘for two’. Each menu item was categorised into one of the 12 food categories that have been defined in a previous research study^32^: appetisers and sides, baked Goods, beverages, burgers, desserts, mains, fried potatoes, pizza, salads, sandwiches, soup, toppings and ingredients.

Restaurant chains were ‘café’ if they did not provide table service and full meals, and mostly sold drinks and snacks (eg, Costa Coffee); ‘family/sit-down restaurants’ if the restaurants provided table service (eg, Beefeater); ‘Western-style fast-food’ if they offered quick meals with little or no table service, often served in disposable packaging, and provided more ‘western’ foods such as chips and fried food (eg, McDonald’s); and ‘Asian-style fast-food’ if they provided mostly non-fried Asian-style fast-food such as sushi and noodles (eg, Itsu). All item-level and restaurant-level variables were manually coded by a single researcher in each year. It is a limitation that it was not possible to double-code these variables due to resource constraints.

### Identifying core menu items

We identified core menu items (those present in all three years) using record linkage techniques.^37^ Only menu items collected from the same restaurants were compared which reduced the number of comparisons. We standardised menu item names by removing special characters and converting strings to lower case characters. A similarity score was calculated for each record pair, based on menu item names and measured by the Jaro-Winkler distance.^38^ The similarity score is one in case of complete agreement, and zero for complete disagreement. We filtered to matches with a similarity score greater than 0.9, and the best match for each item was selected.

To validate the accuracy of this approach, we manually matched McDonald’s menu items from 2019 and 2020 and identified 78 matched items. Using the record linkage approach, 68 out of the 78 items (87.18%) were correctly identified. Three different cut-off scores, 0.8, 0.85, and 0.9 were tested, and the cut-off score of 0.9 yielded the least number of incorrect matches while maintaining all 68 true matches (online supplemental materials, table S2). Record linkage was performed in Python (V.3.6.10; Python Software Foundation). Examples of core menu items identified through this process can be found in online supplemental materials, table S3.

### Statistical analysis

We conducted separate analyses for all menu items and core menu items. The outcomes of interest were energy (kcal), salt (g), saturated fat (g) and sugar (g) per serving. Energy and nutrient density were not primary outcomes of interest, due to the fact that serving size or density information was only provided for around half of menu items.

#### All menu items

We used linear mixed regression models with random intercepts to account for clustering within restaurants, for each outcome. We adjusted for item-level covariates (ie, children’s menu item status, shareable, food category) and a restaurant-level covariate (ie, restaurant type).

#### Core menu items

We used linear mixed regression models with random intercepts for each menu item, for each outcome. We adjusted for item-level covariates (ie, children’s menu item status, shareable, food category). As core menu items were from the same restaurants by definition, we did not adjust for restaurant-level covariates.

To further examine trends for different food categories, we added interaction terms for year and food categories to each set of models, and retained them if they were significant. Yearly predicted mean and 95% CIs were calculated. The unit of analysis was menu item. All statistical analyses were performed in R statistical software from December 2020 to May 2021 (V.4.0.2; R Foundation for Statistical Computing, Vienna, Austria).

### Sensitivity analyses

We conducted several additional analyses to test the robustness of our findings to key methodological assumptions, including using nutrient densities as a complementary set of outcomes (online supplemental materials, table S4). To examine if trends in nutrients were dependent on energy changes, we additionally adjusted nutrient analyses by the energy content of menu items (online supplemental materials, table S5). Also, we repeated our analyses excluding menu items with the largest amounts of energy (ie, top 5% quantile) to minimise the impact of the most extreme portion sizes (online supplemental materials, table S6). We further stratified our analyses by restaurant type to investigate if trends varied (online supplemental materials, tables S7,8). Unadjusted per-item changes were also reported in online supplemental materials, tables S9-12.

## Results

Forty-two restaurants (42.0%) met our inclusion criteria in 2018, 48 restaurants (46.2%) in 2019 and 40 restaurants (40%) in 2020. Twenty-nine restaurants met the inclusion criteria in all three years and were included in the analysis. Among them were six cafés, eight Western-style fast-food restaurants, two Asian-style fast-food restaurants, and 13 family/sit-down restaurants.

### Menu item characteristics

Table 1 shows the characteristics of the 23911 menu items served by these 29 restaurants from 2018 to 2020, respectively. Overall, 31.44% of menu items were from a café, 28.95% from a Western-style fast-food restaurant, 2.89% from an Asian-style fast-food restaurant, and 36.72% from a family/sit-down restaurant. Menu items were predominantly non-sharable (98.16%) and were not described as being for children (95.27%). Beverages and pizza were the two largest categories, comprising 27.14% and 17.82% of all menu items respectively. The characteristics of menu items also varied by year.

**Table 1:** Characteristics of menu items included from 2018 to 2020

	Menu items available in 2018; n (%); (N=7770)	Menu items available in 2019; n (%); (N=9213)	Menu items available in 2020; n (%); (N=6928)	All menu items; n (%); (N=23 911)	Core menu items; n (%); (N=1855)
Type of restaurant					
Cafe	2578 (33.18)	3169 (34.40)	1770 (25.55)	7517 (31.44)	339 (18.27)
Western-style fast-food	2334 (30.04)	2370 (25.72)	2219 (32.03)	6923 (28.95)	931 (50.18)
Asian-style fast-food	202 (2.6)	309 (3.35)	180 (2.60)	691 (2.89)	58 (3.12)
Family/sit-down	2656 (34.18)	3365 (36.52)	2759 (39.82)	8780 (36.72)	527 (28.4)
Shareable					
Non-shareable	7585 (97.62)	9085 (98.61)	6801 (98.17)	23 471 (98.16)	1833 (98.81)
Shareable	185 (2.38)	128 (1.39)	127 (1.83)	440 (1.84)	22 (1.19)
Kids					
Non children’s menu Item	7380 (94.98)	8765 (95.14)	6634 (95.76)	22 779 (95.27)	1820 (98.11)
Children’s menu item	390 (5.02)	448 (4.86)	294 (4.24)	1132 (4.73)	35 (1.89)
Food category					
Appetisers and sides	723 (9.31)	888 (9.64)	705 (10.18)	2316 (9.69)	182 (9.81)
Baked goods	283 (3.64)	539 (5.85)	356 (5.14)	1178 (4.93)	105 (5.66)
Beverages	2112 (27.18)	2600 (28.22)	1778 (25.66)	6490 (27.14)	265 (14.29)
Burgers	300 (3.86)	334 (3.63)	216 (3.12)	850 (3.55)	67 (3.61)
Desserts	541 (6.96)	540 (5.86)	455 (6.57)	1536 (6.42)	93 (5.01)
Mains	864 (11.12)	1195 (12.97)	833 (12.02)	2892 (12.09)	182 (9.81)
Fried potatoes	109 (1.40)	138 (1.50)	136 (1.96)	383 (1.60)	28 (1.51)
Pizza	1493 (19.21)	1446 (15.70)	1323 (19.10)	4262 (17.82)	709 (38.22)
Salads	206 (2.65)	270 (2.93)	157 (2.27)	633 (2.65)	35 (1.89)
Sandwiches	540 (6.95)	637 (6.91)	362 (5.23)	1539 (6.44)	64 (3.45)
Soups	76 (0.98)	97 (1.05)	76 (1.10)	249 (1.04)	15 (0.81)
Toppings and ingredients	523 (6.73)	529 (5.74)	531 (7.66)	1583 (6.62)	110 (5.93)

A total of 1855 (23.27%) core menu items were identified. Over half of core menu items were from Western-style fast-food restaurants (50.18%). Similar to the characteristics of all menu items, core menu items were predominantly non-shareable (98.81%) and not described as being for children (98.11%). Beverages (14.29%) and pizzas (38.22%) were, again, the two largest categories by proportion.

### Trend analysis for all menu items

**Figure 2:**
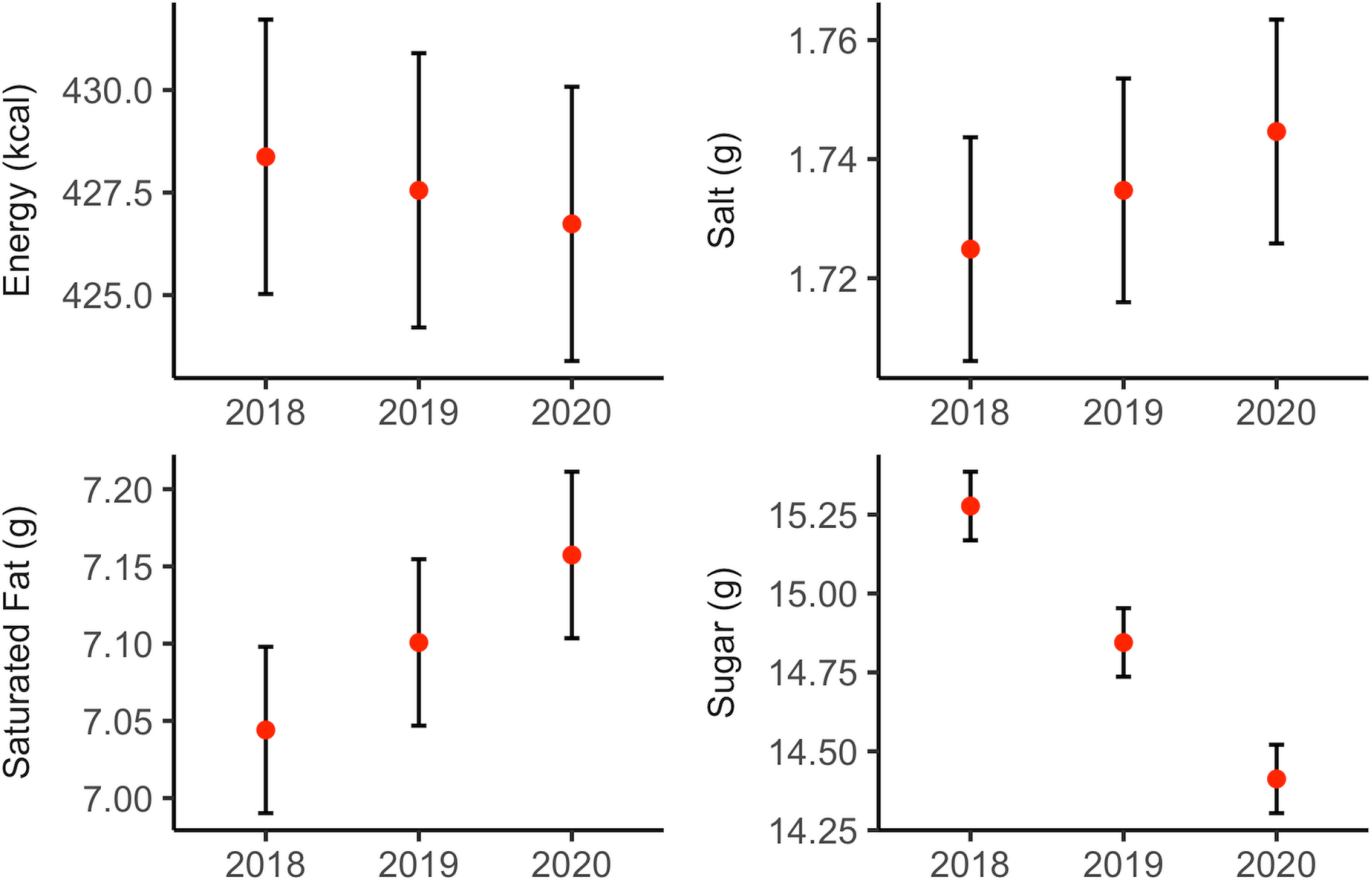
Trends of energy and nutrients per serving, all menu items.

Figure 2 shows the predicted energy (kcal), salt (g), sugar (g) and saturated fat (g) per serving in all menu items, from 2018 to 2020, adjusted for item and restaurant level covariates. Sugar per serving reduced from 15.28 g in 2018 to 14.41 g in 2020 (−5.70%, p<0.05). We also observed a downward trend for energy, and upward trends for salt and saturated fat, but these were not statistically significant. On average, sugar per serving decreased by 0.43 g (95% CI −0.66 to –0.21) per-item per-year in all menu items.

### Trend analysis for all menu items, by food category

**Figure 3:**
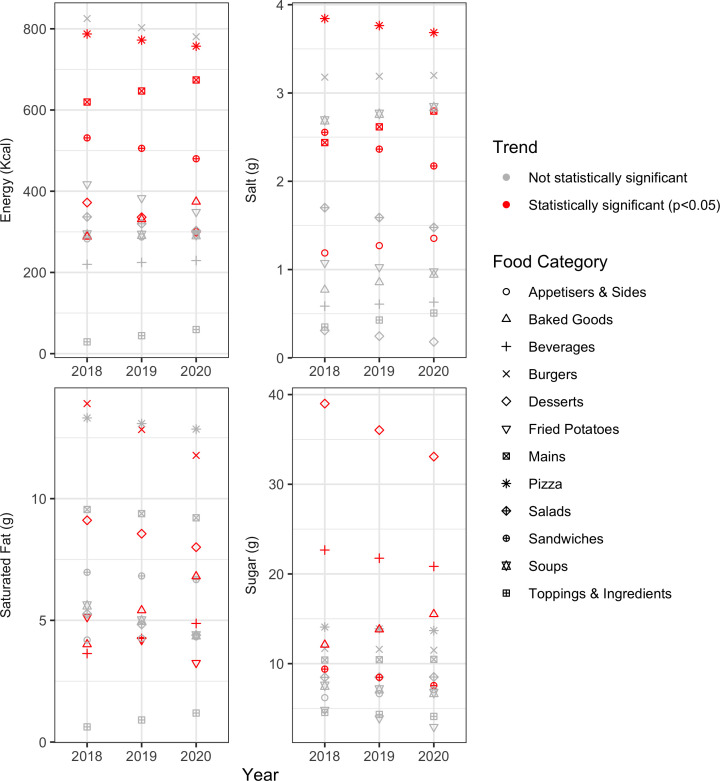
Trends of energy and nutrient per serving, all menu items, by food category.

As shown in figure 3, energy (per-item per-year change: −25.68 kcal, overall percentage change 2018–2020: −9.67%), salt (−0.19 g, −14.92%), and sugar (−0.92 g, −19.50%) content declined in sandwiches. Energy (−15.07kcal, −3.83%) and salt (−0.08 g, −4.13%) content decreased in pizza items, yet both increased in mains (27.15kcal, 8.76%; 0.18 g, 14.61%). The energy (−36.08 kcal, −19.40%), sugar (−2.95 g, −15.15%) and saturated fat (−0.55 g, −12.13%) content reduced in desserts, but increased in baked goods (43.13 kcal, 29.96%; 1.71 g, 28.20%; 1.40 g, 69.56%). The saturated fat content decreased in fried potatoes (−0.94 g, −36.68%) and burgers (−1.06 g, −15.30%). We also observed a small increase in the salt content of appetisers & sides (0.08 g, 13.91%). Among beverages, the sugar content decreased (−0.91 g, −8.05%), but the saturated fat content increased (0.62 g, 34.01%). All of these trends described above were significant (p<0.05). No significant changes were observed in the energy and nutrient content of salads, soups, and toppings and ingredients.

### Trend analysis for core menu items

**Figure 4:**
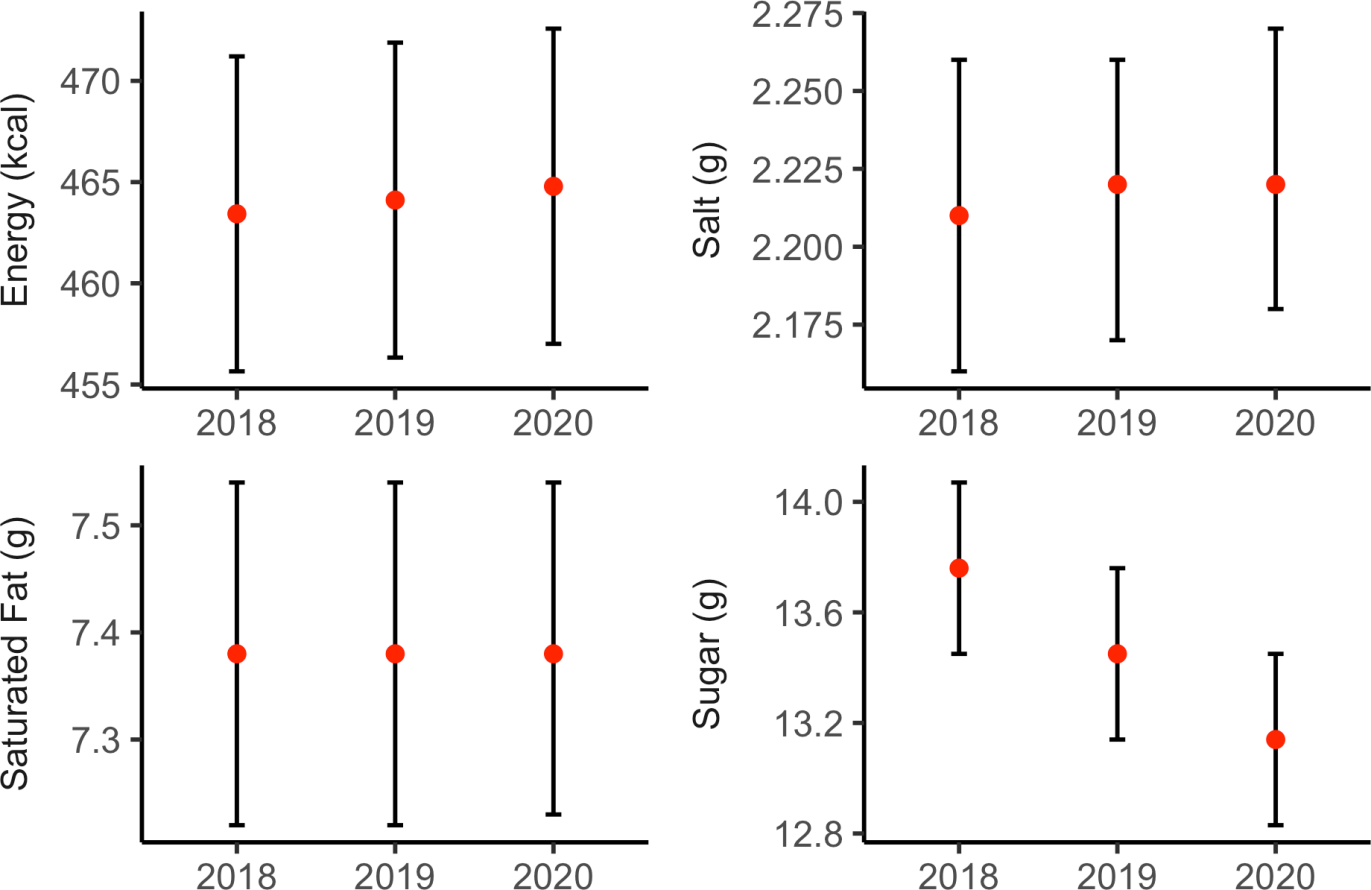
Trends of energy and nutrients per serving, core menu items.

Similar to the results for all menu items, we observed a significant reduction in sugar per serving in core menu items (−4.51%, p<0.05, figure 4). Sugar per serving decreased by 0.31 g per-item per-year (95% CI −0.45 to –0.17). There were no significant changes in salt, fat, and saturated fat content among core menu items.

### Trend analysis for core menu items, by food category

**Figure 5:**
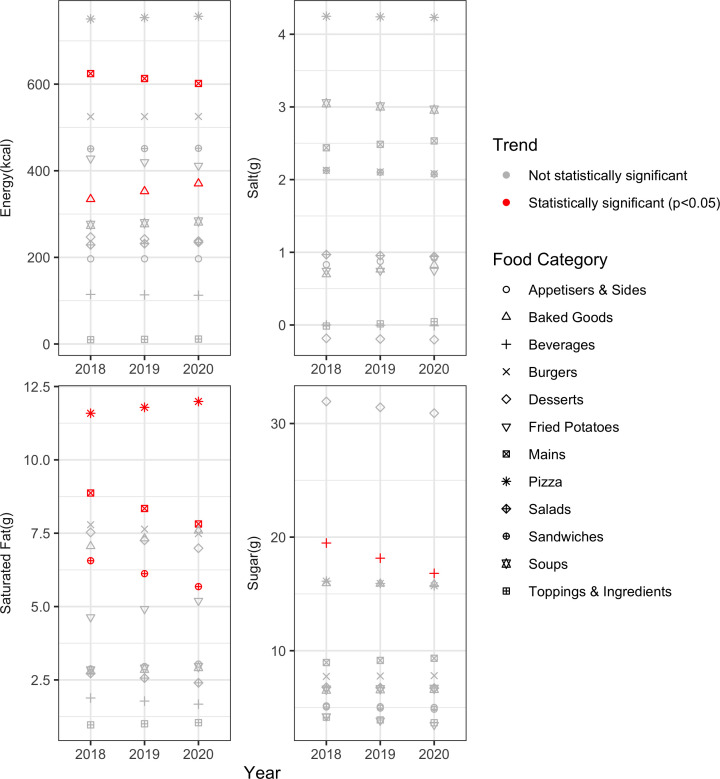
Trends of energy and nutrient per serving, core menu items, by food category.

Among core menu items, energy (−11.39 kcal, −3.65%) and saturated fat (−0.53 g, −11.90%) both decreased in mains (figure 5). Saturated fat content of menu items decreased in sandwiches (−0.44 g, −13.46%), but increased in pizzas (0.20 g, 3.47%). There was a downward trend for sugar in beverage items (−1.33 g, −13.70%), but no significant trend in salt in any food category. Energy increased in baked goods (18.04 kcal, 10.79%). There were no significant changes in appetisers and sides, burgers, desserts, fried potatoes, salads, soups, and toppings and ingredients, among core menu items.

## Discussion

### Summary

This is the first study to evaluate longitudinal trends in energy and nutrient content of items on the menus of large chain restaurants in the UK. In large chain restaurants that consistently provided online nutritional information on menu items in 2018–2020, we studied trends in both all menu items available and core menu items available in all three years. Our results showed that sugar content of all menu items declined from 2018 to 2020. This reduction in sugar was evident in beverages, sandwiches and desserts. Among core menu items, sugar content reduced significantly from 2018 to 2020, especially in beverages. The magnitude of sugar reduction was smaller in core menu items compared with all menu items (0.31 g/year vs 0.43 g/year). Energy, salt and saturated fat content in menu items remained constant overall, despite some food categories showing increasing (eg, energy, sugar and saturated fat content increased in baked goods) or decreasing (eg, energy, salt and sugar reduction in sandwiches) trends. Fewer food categories had significant changes in energy, sugar, salt and saturated fat content among core menu items, compared with all menu items available.

### Strengths and limitations

We captured data on the top UK chain restaurants based on either number of outlets or sales, and restricted our analyses to the same 29 restaurants in all three years. The longitudinal nature of our data collection allowed assessment of trends over time. We also used record linkage techniques to identify core menu items, allowing us to suggest whether reformulation had occurred during this time period. The data collected for this analysis form, in their own right, a valuable resource for future policy evaluation.

However, this study is not without its limitations. First, our data only included items on online menus. It is possible that menu items provided online and in-store differ. However, it has been estimated that 85% of products found in-store in supermarkets are available online, indicating that online information is a good proxy for packaged products in physical stores.^39^ Further validation work is needed to investigate similarities between online and in-store restaurant menus. Second, online nutritional information can be inaccurate. Nevertheless, we made efforts to identify and remove data errors through cleaning. Additionally, the 2018 and 2019 data were collected in March - April, whereas the 2020 data were collected in October–November. Seasonal variations may introduce bias to our analyses. In all our models, we adjusted for item-level covariates to minimise the potential impact of seasonal variation on menus. Furthermore, our results based on core menu items, which are unlikely to be affected by season as they are available all year round, were consistent with the results from all menu items. Lastly, this remains an analysis of three-year change among 29 large chain restaurants. We do not know how representative these changes are of those in other chain restaurants, or the out-of-home market in general. Nonetheless, the government’s consultation response on calorie labelling published in 2018 indicates that large businesses (eg, large chain restaurants) make up nearly half of all out-of-home food and drink sales in the UK.^26^ Future research should continue to assess nutritional changes in restaurant menu items, including items offered by other large chain restaurants and small independent retailers not included here, and over a longer term.

### Lessons for policy and practice

Our finding that the sugar content of menu items served by large UK chain restaurants decreased over time may reflect the fact that sugar has received a considerable amount of policy attention in recent years in the UK.^22 40^ In 2016, the government challenged the food and hospitality industry to reduce sugar in many product categories.^41^ In April 2018, the UK implemented the soft drinks industry levy (SDIL), aimed at reducing the sugar content of sugar-sweetened beverages by incentivising companies to reformulate.^40^ Our findings indicate that the change in sugar content of core menu items was particularly driven by sugar reduction in beverages, which mirrors the direction of observed effect of the SDIL on the UK soft drink market.^42^ As beverages available on restaurant menus are often supplied by large manufacturers (eg, Coca-Cola or PepsiCo) rather than produced by chain restaurants themselves, the sugar reduction in beverages we observed may be more attributable to changes made by soft drink manufacturers than large chain restaurants.

Studies in the USA suggest that the energy and nutrient content of core menu items has changed little over time, but in recent years, there has been a decline in energy and sodium for newly introduced items.^14 15 43 44^ Such changes in the USA may reflect the effects of mandatory calorie menu labelling in large chain restaurants, which went into effect in 2018, and has been previously found to be associated with small reductions in energy content; and the voluntary sodium reduction guidance for industry (including the out-of-home sector), which was published in 2016.^45–47^ In the UK, we found that the sugar content of core menu items also reduced between 2018 and 2020. It is plausible that fiscal policies such as the SDIL have incentivised more reformulation than voluntary reduction programmes or nutrition labelling rules. Additionally, we found fewer food categories with significant changes in energy, salt, sugar, and saturated fat content among core menu items, compared with all menu items. This is consistent with the hypothesis that core menu items may be less likely to undergo reformulation.

It has been established that a reduction in sugar consumption, especially sugar-sweetened beverages, can contribute to positive health outcomes.^48–53^ Given the frequency with which people eat outside the home, the observed decline in sugar content in items served by large chain restaurants may result in health improvements at the population level.^6^ However, it does not necessarily imply a reduction in overall population sugar intake. Our results were not weighted by sales volume and we did not include data on dietary intake.

Policies focusing on single nutrients do not necessarily improve the overall nutrient quality of restaurant food items. As an example, to achieve energy reduction by reformulation, more salt may be required to maintain consumer acceptability.^54^ Our results suggest that while the sugar content reduced significantly in restaurant menu items in 2018 - 2020, other nutrients, such as salt and saturated fat, remained unchanged. The salt reduction programme in the UK has been broadly successful, with population salt intake in the UK reduced by 15% from 2003/2004 to 2011.^55^ However, our findings indicate that this progress may have stagnated in certain categories for the out-of-home sector. This finding is consistent with reports elsewhere.^24^ The salt content of appetisers and sides and mains increased between 2018 and 2020, implying a possible rebound in salt density or/and an increase in portion sizes.

Despite some reductions in the sugar content of menu items, the magnitude of sugar reduction we observed is far from the 20% reduction target by 2020 in relevant categories, which was set by the voluntary sugar reduction programme in 2016.^22^ In addition, energy, salt and saturated fat content did not change significantly in restaurant menu items. Overall, this signals that little progress has been made towards a healthier restaurant environment through voluntary policies between 2018 and 2020, when no mandatory policy for the out-of-home sector was implemented. More robust policy approaches, such as mandatory menu labelling, granting of planning permission to new food outlets based on menu healthiness, reformulation taxes, and restricting advertising to items with particular nutritional contents, may improve the overall nutritional quality of restaurant foods.^28 56–59^

### Conclusion

From 2018 to 2020, the energy, salt and saturated fat content of items served by 29 UK large chain restaurants remained constant, while the sugar content declined. This reduction in sugar was observed in all menu items as well as those present in all three years, yet the magnitude of the reduction was smaller among the latter and particularly marked in beverages. This may reflect a response to the government’s sugar reduction strategy and SDIL. Future policies addressing the overall nutritional quality of restaurant foods, rather than single nutrients, may help the restaurant sector move towards offering healthier foods.

## Data Availability

Data are available on reasonable request. Anonymised dataset used in the analyses are available on request. Please contact the authors via email if you believe the data would be of value to your research. Use of our data is only permitted for non-commercial purposes.

## References

[1] Muc M , Jones A , Roberts C , et al . A bit or a lot on the side? Observational study of the energy content of starters, sides and desserts in major UK restaurant chains. BMJ Open 2019;9:e029679. 10.1136/bmjopen-2019-029679 PMC6797243 31594875

[2] Jaworowska A , Blackham T , Davies IG , et al . Nutritional challenges and health implications of takeaway and fast food. Nutr Rev 2013;71:310–8. 10.1111/nure.12031 23590707

[3] Robinson E , Jones A , Whitelock V , et al . (Over)eating out at major UK restaurant chains: observational study of energy content of main meals. BMJ 2018;363:k4982. 10.1136/bmj.k4982 30541906 PMC6290483

[4] Young M , Coppinger T , Reeves S . The nutritional value of children's menus in chain restaurants in the United Kingdom and Ireland. J Nutr Educ Behav 2019;51:817–25. 10.1016/j.jneb.2019.04.018 31126724

[5] Roberts SB , Das SK , Suen VMM , et al . Measured energy content of frequently purchased restaurant meals: multi-country cross sectional study. BMJ 2018;363:k4864. 10.1136/bmj.k4864 30541752 PMC6290458

[6] Adams J , Goffe L , Brown T , et al . Frequency and socio-demographic correlates of eating meals out and take-away meals at home: cross-sectional analysis of the UK national diet and nutrition survey, waves 1-4 (2008-12). Int J Behav Nutr Phys Act 2015;12:51. 10.1186/s12966-015-0210-8 25889159 PMC4404110

[7] Guthrie JF , Lin B-H , Frazao E . Role of food prepared away from home in the American diet, 1977-78 versus 1994-96: changes and consequences. J Nutr Educ Behav 2002;34:140–50. 10.1016/S1499-4046(06)60083-3 12047838

[8] Orfanos P , Naska A , Trichopoulos D , et al . Eating out of home and its correlates in 10 European countries. The European prospective investigation into cancer and nutrition (EPIC) study. Public Health Nutr 2007;10:1515–25. 10.1017/S1368980007000171 17582244

[9] Rosenheck R . Fast food consumption and increased caloric intake: a systematic review of a trajectory towards weight gain and obesity risk. Obes Rev 2008;9:535–47. 10.1111/j.1467-789X.2008.00477.x 18346099

[10] Bezerra IN , Curioni C , Sichieri R . Association between eating out of home and body weight. Nutr Rev 2012;70:65–79. 10.1111/j.1753-4887.2011.00459.x 22300594

[11] Goffe L , Rushton S , White M , et al . Relationship between mean daily energy intake and frequency of consumption of out-of-home meals in the UK national diet and nutrition survey. Int J Behav Nutr Phys Act 2017;14:131. 10.1186/s12966-017-0589-5 28938893 PMC5610411

[12] Freisling H , Viallon V , Lennon H , et al . Lifestyle factors and risk of multimorbidity of cancer and cardiometabolic diseases: a multinational cohort study. BMC Med 2020;18:5. 10.1186/s12916-019-1474-7 31918762 PMC6953215

[13] Singh GM , Danaei G , Farzadfar F , et al . The age-specific quantitative effects of metabolic risk factors on cardiovascular diseases and diabetes: a pooled analysis. PLoS One 2013;8:e65174. 10.1371/journal.pone.0065174 23935815 PMC3728292

[14] Bleich SN , Soto MJ , Dunn CG , et al . Calorie and nutrient trends in large U.S. chain restaurants, 2012-2018. PLoS One 2020;15:e0228891. 10.1371/journal.pone.0228891 32040526 PMC7010289

[15] Bleich SN , Wolfson JA , Jarlenski MP . Calorie changes in large chain restaurants from 2008 to 2015. Prev Med 2017;100:112–6. 10.1016/j.ypmed.2017.04.004 28389331

[16] Eyles H , Jiang Y , Blakely T , et al . Five year trends in the serve size, energy, and sodium contents of New Zealand fast foods: 2012 to 2016. Nutr J 2018;17:65. 10.1186/s12937-018-0373-7 29983114 PMC6036696

[17] Scourboutakos MJ , Orr S , Hobin E , et al . Assessing the early impact of menu-labeling on calories in chain restaurants in Ontario, Canada. Am J Prev Med 2019;56:e195–203. 10.1016/j.amepre.2019.01.017 31104725

[18] Theis DRZ , White M . Is obesity policy in England fit for purpose? Analysis of government strategies and policies, 1992-2020. Milbank Q 2021;99:126–70. 10.1111/1468-0009.12498 33464689 PMC7984668

[19] Department of Health and Social Care . Public health responsibility deal 2011. Available: https://www.gov.uk/government/news/public-health-responsibility-deal [Accessed 15 Apr 2021].

[20] Public Health England . Salt reduction: targets for 2017, 2017. Available: https://www.gov.uk/government/publications/salt-reduction-targets-for-2017 [Accessed 12 Mar 2021].

[21] Public Health England . Calorie reduction: guidelines for the food industry 2020. Available: https://www.gov.uk/government/publications/calorie-reduction-guidelines-for-the-food-industry [Accessed 15 Apr 2021].

[22] Public Health England . Sugar reduction programme 2018. Available: https://publichealthengland.exposure.co/sugar-reduction-programme [Accessed 12 Mar 2021].

[23] Public Health England . Sugar reduction: report on progress between 2015 and 2019, 2020. Available: https://assets.publishing.service.gov.uk/government/uploads/system/uploads/attachment_data/file/925027/SugarReportY3.pdf [Accessed 19 Jan 2021].

[24] Public Health England . Salt targets 2017: second progress report 2020. Available: https://assets.publishing.service.gov.uk/government/uploads/system/uploads/attachment_data/file/915371/Salt_targets_2017_Second_progress_report_031020.pdf [Accessed 13 Jun 2021].

[25] Tackling obesity: empowering adults and children to live healthier lives, 2020. Available: https://www.gov.uk/government/publications/tackling-obesity-government-strategy/tackling-obesity-empowering-adults-and-children-to-live-healthier-lives [Accessed 31 Oct 2020].

[26] Department of Health and Social Care . Calorie labelling for food and drink served outside of the home 2018. Available: https://www.gov.uk/government/consultations/calorie-labelling-for-food-and-drink-served-outside-of-the-home [Accessed 15 Feb 2021].

[27] Department of Health and Social Care . Childhood obesity: a plan for action, chapter 2 2018. Available: https://www.gov.uk/government/publications/childhood-obesity-a-plan-for-action-chapter-2 [Accessed 12 Mar 2021].

[28] Department of Health and Social Care; Jo Churchill MP . Calorie labelling on menus to be introduced in cafes, restaurants and takeaways 2021 [Government renews drive to tackle obesity and improve the nation’s health]. Available: https://www.gov.uk/government/news/calorie-labelling-on-menus-to-be-introduced-in-cafes-restaurants-and-takeaways [Accessed 2 Jun 2021].

[29] Parliament U . Calorie labelling (out of home sector) (England) regulations 2021, 2021. Available: https://statutoryinstruments.parliament.uk/details/uFGyH5l4/SI-2021/ [Accessed 13 Jun 2021].

[30] Technomic . Technomic top 100 U.K. chain restaurant report 2014. Available: https://www.technomic.com/available-studies/industry-reports [Accessed 2 Dec 2020].

[31] Robinson E , Burton S , Gough T , et al . Point of choice kilocalorie labelling in the UK eating out of home sector: a descriptive study of major chains. BMC Public Health 2019;19:649. 10.1186/s12889-019-7017-5 31138179 PMC6540449

[32] Theis DRZ , Adams J . Differences in energy and nutritional content of menu items served by popular UK chain restaurants with versus without voluntary menu labelling: a cross-sectional study. PLoS One 2019;14:e0222773 https://journals.plos.org/plosone/article?id=10.1371/journal.pone.0222773 10.1371/journal.pone.0222773 31618202 PMC6795485

[33] Import.io: mission-critical web data. Available: https://www.import.io [Accessed 24 Nov 2021].

[34] Scrapy at a glance. Available: https://docs.scrapy.org/en/latest/intro/overview.html [Accessed 2 Dec 2020].

[35] Camelot . PDF table extraction for humans. Available: https://camelot-py.readthedocs.io/en/master/ [Accessed 15 Apr 2021].

[36] tabula-py . Read tables in a PDF into DataFrame. Available: https://tabula-py.readthedocs.io/en/latest/ [Accessed 15 Apr 2021].

[37] Christen P . Data matching: concepts and techniques for record linkage, entity resolution, and duplicate detection. Springer Science & Business Media, 2012.

[38] Springer International Publishing . Efficient approximate entity matching using Jaro-Winkler distance. Cham, 2017.

[39] Bhatnagar P , Scarborough P , Kaur A , et al . Are food and drink available in online and physical supermarkets the same? A comparison of product availability, price, price promotions and nutritional information. Public Health Nutr 2021;24:819–25. 10.1017/S1368980020004346 33109282 PMC7610557

[40] HM treasury. Soft drinks industry levy comes into effect, 2018. Available: https://www.gov.uk/government/news/soft-drinks-industry-levy-comes-into-effect [Accessed 12 Mar 2021].

[41] Cabinet Office, Department of Health and Social Care, HM Treasury . Childhood obesity: a plan for action 2016. Available: https://www.gov.uk/government/publications/childhood-obesity-a-plan-for-action [Accessed 12 Mar 2021].

[42] Scarborough P , Adhikari V , Harrington RA , et al . Impact of the announcement and implementation of the UK soft drinks industry levy on sugar content, price, product size and number of available soft drinks in the UK, 2015-19: a controlled interrupted time series analysis. PLoS Med 2020;17:e1003025. 10.1371/journal.pmed.1003025 32045418 PMC7012398

[43] Bleich SN , Wolfson JA , Jarlenski MP . Calorie changes in large chain restaurants: declines in new menu items but room for improvement. Am J Prev Med 2016;50:e1–8. 10.1016/j.amepre.2015.05.007 26163168 PMC4691555

[44] Wolfson JA , Moran AJ , Jarlenski MP , et al . Trends in sodium content of menu items in large chain restaurants in the U.S. Am J Prev Med 2018;54:28–36. 10.1016/j.amepre.2017.08.018 29056370

[45] U.S. Food and Drug Administration . Draft guidance for industry: target mean and upper bound concentrations for sodium in commercially processed, packaged, and prepared foods for voluntary sodium reduction goals U.S. food and drug administration, 2016. Available: https://www.fda.gov/regulatory-information/search-fda-guidance-documents/draft-guidance-industry-target-mean-and-upper-bound-concentrations-sodium-commercially-processed [Accessed 12 Jan 2021].

[46] Bleich SN , Economos CD , Spiker ML , et al . A systematic review of calorie labeling and modified calorie labeling interventions: impact on consumer and restaurant behavior. Obesity 2017;25:2018–44. 10.1002/oby.21940 29045080 PMC5752125

[47] Food and Drug Administration, HHS . Food labeling; nutrition labeling of standard menu items in restaurants and similar retail food establishments. final rule. Fed Regist 2014;79:71155–259. 25438344

[48] Johnson RJ , Sánchez-Lozada LG , Andrews P , et al . Perspective: a historical and scientific perspective of sugar and its relation with obesity and diabetes. Adv Nutr 2017;8:412–22. 10.3945/an.116.014654 28507007 PMC5421126

[49] Manyema M , Veerman LJ , Chola L , et al . The potential impact of a 20% tax on sugar-sweetened beverages on obesity in South African adults: a mathematical model. PLoS One 2014;9:e105287. 10.1371/journal.pone.0105287 25136987 PMC4138175

[50] Hu FB . Resolved: there is sufficient scientific evidence that decreasing sugar-sweetened beverage consumption will reduce the prevalence of obesity and obesity-related diseases. Obes Rev 2013;14:606–19. 10.1111/obr.12040 23763695 PMC5325726

[51] Bernabé E , Vehkalahti MM , Sheiham A , et al . Sugar-sweetened beverages and dental caries in adults: a 4-year prospective study. J Dent 2014;42:952–8. 10.1016/j.jdent.2014.04.011 24813370

[52] Asgari-Taee F , Zerafati-Shoae N , Dehghani M , et al . Association of sugar sweetened beverages consumption with non-alcoholic fatty liver disease: a systematic review and meta-analysis. Eur J Nutr 2019;58:1759–69. 10.1007/s00394-018-1711-4 29761318

[53] Pase MP , Himali JJ , Beiser AS , et al . Sugar- and artificially sweetened beverages and the risks of incident stroke and dementia: a prospective cohort study. Stroke 2017;48:1139–46. 10.1161/STROKEAHA.116.016027 28428346 PMC5405737

[54] Leshem M . Biobehavior of the human love of salt. Neurosci Biobehav Rev 2009;33:1–17. 10.1016/j.neubiorev.2008.07.007 18708089

[55] He FJ , Brinsden HC , MacGregor GA . Salt reduction in the United Kingdom: a successful experiment in public health. J Hum Hypertens 2014;28:345–52. 10.1038/jhh.2013.105 24172290

[56] Seferidi P , Millett C , Laverty AA . Industry self-regulation fails to deliver healthier diets, again. BMJ 2021;372:m4762. 10.1136/bmj.m4762 33408098

[57] Department of Health and Social Care, Department of Digital C, Media & Sport . Introducing further advertising restrictions on TV and online for products high in fat, sugar and salt (HFSS) 2019.

[58] National Food Strategy . Recommendations in full, 2021. Available: https://www.nationalfoodstrategy.org [Accessed 24 Nov 2021].

[59] Public Health England . Healthy weight environments: using the planning system, 2020. 10.1016/j.puhip.2020.100007 PMC7194517 34171040

