# Supplementary material for: Trends in energy and nutrient content of menu items served by large UK chain restaurants from 2018 to 2020: an observational study
Source: BMJ Open. 2021 Dec 30;11(12):e054804. doi: 10.1136/bmjopen-2021-054804 (PMC8718418; doi:10.1136/bmjopen-2021-054804)
Supplement: Supplementary data [file bmjopen-2021-054804supp001.pdf]

BMJ Publishing Group Limited (BMJ) disclaims all liability and responsibility arising from any reliance placed on this supplemental material which has been supplied by the author(s)

SUPPLEMENTARY MATERIALS

Trends in energy and nutrient content of menu items served by large UK chain restaurants from 2018 to 2020: an observational study

Table S1 List of Chain Restaurants Included in Our Study

These restaurants provided nutritional information online in all of 2018, 2019, and 2020.

All Bar One
ASK Italian
Beefeater Grill
Brewers Fayre
Burger King
Caffe Nero
Chef & Brewer Pub Co.
Costa Coffee
Domino's Pizza
Flaming Grill Pub Co.
Gourmet Burger Kitchen
Greggs
Itsu
KFC
McDonalds UK
Nando’s
Papa John's
PAUL
Pizza Hut
Pizza Express
Pret A Manger
Starbucks
Subway
Table Table
Toby Carvery
Wagamama
Wetherspoon
Wimpy
YO! Sushi

Table S2 Record Linkage Using Different Cut-off Points for the Record Linkage Process

Cut-off [a]	Correctly identified matches Total: 78	Incorrectly identified matches
0.80	68	2
0.85	68	1
0.90	68	0

[a] Three different cut-off scores – 0.80, 0.85, and 0.90 – were tested using McDonald's 2019 and 2020 menu data. All three cut-off scores identified 68 correct matches, but the incorrectly identified match was zero when the threshold of 0.90 was chosen.

Table S3 Examples of Core Menu Items Identified Through the Record Linkage Process

Restaurant Name	Item Name (2018)	Item Name (2019)	Item Name (2020)
Wagamama	chicken & prawn pad thai	Chicken & Prawn Pad Thai	chicken + prawn pad-thai
McDonalds UK	Chicken McNuggets 6	Chicken Mcnuggets, 6	Chicken McNuggets (6 pieces)
Itsu	Chicken thai rice bowl	Thai Chicken Rice Bowl	thai chicken riceâ€™bowl
Greggs	Sugar Str& Doughnut	Sugar Strand Doughnut	Sugar Strand Doughnut

Table S4 Trends in Energy and Nutrient Density (N = 12,181)

Ten restaurants that provided serving weight in all three years were selected for the trend analysis. **These ten restaurants were**: ASK Italian, Costa Coffee, Gourmet Burger Kitchen, Greggs, Papa John's, PAUL, Pizza Hut, Pizza Express, and Starbucks.

A total of 12,181 items with serving weights from these ten restaurants were included in the analysis. We used linear mixed regression models with random intercepts to account for clustering within restaurants, for each outcome. We adjusted for item-level covariates (i.e., children's menu item status, shareable, food category) and a restaurant-level covariate (i.e., restaurant type).

	Predicted Mean (95%CI) [a] **2018** **N= 4,175**	Predicted Mean (95%CI) [a] **2019** **N = 4,768**	Predicted Mean (95%CI) [a] **2020** **N = 3,238**	Trend (P-Value)
Energy (kcal/100g)	207.59(205.59, 209.59)	208.62(206.62, 210.62)	209.65(207.65, 211.64)	0.40
Salt (g/100g)	0.95(0.94, 0.96)	0.94(0.92, 0.95)	0.92(0.91, 0.94)	0.56
Sugar (g/100g)	8.17(8.04, 8.30)	7.85(7.72, 7.98)	7.54(7.41, 7.67)	<0.05
Saturated Fat (g/100g)	3.47(3.43, 3.51)	3.63(3.59, 3.67)	3.80(3.76, 3.84)	<0.05

[a] Linear mixed models adjusted for item-level covariates (i.e., children's menu item status, shareable, food category) and a restaurant-level covariate (i.e., restaurant type).

Table S5 Trends in Energy and Nutrient Values, Adjusting for Energy (N = 23,911)

We ran a set of linear mixed models using all menu items (N = 23,911), adjusting for item-level covariates (i.e., children's menu item status, shareable, food category), a restaurant-level covariate (i.e., restaurant type), and **energy**.

	Predicted Mean (95%CI) [a] **2018** **N = 7,770**	Predicted Mean (95%CI) [a] **2019** **N = 9,213**	Predicted Mean (95%CI) [a] **2020** **N = 6,928**	Trend (P-Value)
Salt (g)	1.71(1.69, 1.73)	1.73(1.71, 1.75)	1.75(1.73, 1.78)	**<.05**
Sugar (g)	15.20(15.06, 15.33)	14.85(14.72, 14.99)	14.51(14.37, 14.64)	**<.05**
Saturated Fat (g)	6.98(6.89, 7.06)	7.11(7.02, 7.19)	7.23(7.15, 7.32)	**<.05**

[a] Liner-mixed models adjusted for energy, item-level covariates (i.e., children's menu item status, shareable, food category) and a restaurant-level covariate (i.e., restaurant type)

Table S6 Trends in Energy and Nutrient Per Serving, Excluding High Calorie Items (top 5%) (N = 21,743)

We excluded 2,168 menu items that contained higher than 1086 kcal (top 5%) in our analysis. A total of 21,743 items were included for this analysis. We ran a set of linear mixed models, adjusting for item-level covariates (i.e., children's menu item status, shareable, food category) and a restaurant-level covariate (i.e., restaurant type).

	Predicted Mean (95%CI) [a] **2018** **N = 7,324**	Predicted Mean (95%CI) [a] **2019** **N = 8,618**	Predicted Mean (95%CI) [a] **2020** **N = 5,801**	Trend (P-Value)
Energy (kcal)	372.71(370.08, 375.33)	372.03(369.41, 374.65)	371.35(368.73, 373.98)	0.67
Salt (g)	1.54(1.53, 1.56)	1.53(1.52, 1.55)	1.52(1.51, 1.54)	0.34
Sugar (g)	14.93(14.82, 15.04)	14.46(14.35, 14.57)	13.99(13.88, 14.10)	<0.05
Saturated Fat (g)	6.05(6.01, 6.09)	6.17(6.13, 6.21)	6.29(6.25, 6.33)	<0.05

[a] Linear mixed models adjusted for item-level covariates (i.e., children's menu item status, shareable, food category) and a restaurant-level covariate (i.e., restaurant type)

Table S7 Predicted Mean Energy and Nutrients Per Serving among All Menu Items, by Restaurant Type (N = 23,911)

Type of Restaurant	Predicted Mean (95%CI) [a] **2018** **N = 7,770**	Predicted Mean (95%CI) [a] **2019** **N = 9,213**	Predicted Mean (95%CI) [a] **2020** **N = 6,928**	**Change/Year**
Energy				
Café	359.24(356.08, 362.41)	366.39(363.22, 369.55)	373.53(370.36, 376.70)	7.14(-1.51, 15.80)
Western-style Fast-Food	384.69(381.52, 387.85)	374.60(371.44, 377.77)	364.51(361.35, 367.68)	**-10.09(-18.36, -1.81)** [b]
Asian-style Fast-Food	215.97(212.80, 219.13)	313.90(310.74, 317.07)	411.84(408.68, 415.01)	**97.94(69.84, 126.03)** [b]
Family/Sit-down	544.65(541.48, 547.81)	537.88(534.71, 541.04)	531.10(527.94, 534.27)	-6.77(-14.64, 1.10)
Salt				
Café	1.31(1.29, 1.33)	1.34(1.32, 1.35)	1.36(1.35, 1.38)	0.03(-0.02, 0.07)
Western-style Fast-Food	1.81(1.79, 1.82)	1.72(1.71, 1.74)	1.64(1.62, 1.66)	**-0.08(-0.13, -0.04)** [b]
Asian-style Fast-Food	1.25(1.23, 1.27)	1.48(1.46, 1.50)	1.71(1.70, 1.73)	**0.23(0.02, 0.44)** [b]
Family/Sit-down	2.05(2.03, 2.07)	2.12(2.10, 2.14)	2.19(2.18, 2.21)	**0.07(0.03, 0.11)** [b]
Sugar				
Café	14.92(14.81, 15.03)	14.49(14.38, 14.60)	14.06(13.95, 14.17)	**-0.43(-0.66, -0.21)** [b]
Western-style Fast-Food	14.14(14.03, 14.24)	13.70(13.59, 13.81)	13.27(13.16, 13.38)	
Asian-style Fast-Food	12.66(12.55, 12.77)	12.23(12.12, 12.34)	11.79(11.68, 11.90)	
Family/Sit-down	16.75(16.64, 16.86)	16.32(16.21, 16.43)	15.88(15.77, 15.99)	
Saturated Fat				
Café	6.27(6.21, 6.32)	6.94(6.89, 7.00)	7.62(7.57, 7.67)	**0.68(0.46, 0.89)** [b]
Western-style Fast-Food	5.72(5.66, 5.77)	5.63(5.57, 5.68)	5.54(5.49, 5.59)	-0.09(-0.29, 0.12)
Asian-style Fast-Food	3.50(3.45, 3.55)	3.78(3.73, 3.83)	4.06(4.01, 4.11)	0.28(-0.57, 1.13)
Family/Sit-down	9.08(9.02, 9.13)	8.74(8.69, 8.80)	8.41(8.35, 8.46)	**-0.33(-0.53, -0.14)** [b]

[a] Linear mixed models adjusted for children's menu item status, shareable, food category, and restaurant type. Interaction terms of year and restaurant type were retained if significant.
[b] Boldface indicates statistical significance (P<0.05)

Table S8 Predicted Mean Energy and Nutrients Per Serving, Core Menu Items, by Restaurant Type (N = 1,855)

**Type of Restaurant**	Predicted Mean (95%CI) [a] **2018** **N = 1,855**	Predicted Mean (95%CI) [a] **2019** **N= 1,855**	Predicted Mean (95%CI) [a] **2020** **N = 1,855**	**Change/Year**
Energy				
Café	390.34(383.53, 397.16)	389.40(382.58, 396.21)	388.45(381.63, 395.26)	-0.95(3.4,1.5)
Western-style Fast-Food	439.98(433.17, 446.79)	439.03(432.22, 445.84)	438.08(431.27, 444.89)	
Asian-style Fast-Food	317.07(310.26, 323.88)	316.12(309.31, 322.93)	315.17(308.36, 321.98)	
Family/Sit-down	549.47(542.66, 556.28)	548.52(541.71, 555.33)	547.57(540.76, 554.38)	
Salt				
Café	1.65(1.61, 1.69)	1.66(1.62, 1.70)	1.67(1.63, 1.71)	0.01(0.01, 0.03)
Western-style Fast-Food	2.13(2.09, 2.17)	2.13(2.09, 2.18)	2.14(2.10, 2.18)	
Asian-style Fast-Food	1.73(1.69, 1.77)	1.74(1.70, 1.78)	1.75(1.71, 1.79)	
Family/Sit-down	2.42(2.38, 2.46)	2.43(2.39, 2.47)	2.43(2.39, 2.47)	
Sugar				
Café	14.52(14.25, 14.78)	13.64(13.37, 13.90)	12.76(12.49, 13.02)	**-0.88(-1.13, -0.62)** [b]
Western-style Fast-Food	12.67(12.40, 12.94)	12.38(12.12, 12.65)	12.09(11.83, 12.36)	**-0.29(-0.46, -0.11)** [b]
Asian-style Fast-Food	10.60(10.34, 10.87)	10.49(10.23, 10.76)	10.39(10.12, 10.65)	-0.11(-0.92, 0.71)
Family/Sit-down	14.67(14.41, 14.94)	14.74(14.48, 15.01)	14.81(14.55, 15.08)	0.07(-0.14, 0.28)
Saturated Fat				
Café	7.30(7.16, 7.44)	7.37(7.22, 7.51)	7.44(7.29, 7.58)	0.07(-0.08, 0.22)
Western-style Fast-Food	6.26(6.11, 6.40)	6.40(6.25, 6.54)	6.54(6.40, 6.68)	**0.14(0.04, 0.24)** [b]
Asian-style Fast-Food	4.69(4.55, 4.83)	4.80(4.66, 4.94)	4.91(4.77, 5.05)	0.11(-0.38, 0.61)
Family/Sit-down	9.06(8.92, 9.21)	8.83(8.69, 8.97)	8.60(8.46, 8.74)	**-0.23(-0.36, -0.11)** [b]

[a] Linear mixed models adjusted for children's menu item status, shareable, food category, and restaurant type. Interaction terms of year and restaurant type were retained if significant.
[b] Boldface indicates statistical significance (P<0.05)

Table S9 Predicted Mean Energy and Nutrients Per Serving, Unadjusted Models, All Menu Items (N = 23,911)

	Predicted Mean (95%CI) [a] **2018** **N = 7,770**	Predicted Mean (95%CI) [a] **2019** **N = 9,213**	Predicted Mean (95%CI) [a] **2020** **N = 6,928**	Trend (P-Value)
Energy (kcal)	432.79(430.48,435.11)	427.22(424.91,429.54)	421.66(419.34,423.97)	0.05
Salt (g)	1.75(1.73,1.76)	1.73(1.72,1.75)	1.72(1.70,1.73)	0.34
Sugar (g)	15.19(15.14,15.24)	14.85(14.80,14.90)	14.51(14.46,14.56)	<0.05
Saturated Fat (g)	7.09(7.05,7.13)	7.10(7.06,7.14)	7.10(7.06,7.14)	0.94

[a] Crude linear mixed models without adjusting for covariates, all menu items

Table S10 Predicted Mean Energy and Nutrients Per Serving, Unadjusted Models, Core Menu Items (N=1,855)

	Predicted Mean (95%CI) **2018** **N = 1,855**	Predicted Mean (95%CI) **2019** **N = 1,855**	Predicted Mean (95%CI) **2020** **N = 1,855**	Trend (P-Value)
Energy (kcal)	463.33(455.55, 471.11)	464.11(456.33, 471.89)	464.89(457.11, 472.67)	0.57
Salt (g)	2.21(2.16, 2.26)	2.22(2.17, 2.26)	2.23(2.18, 2.27)	0.33
Sugar (g)	13.76(13.45-14.07)	13.45(13.14-13.76)	13.14(12.82-13.45)	<0.05
Saturated Fat (g)	7.38(7.22, 7.54)	7.38(7.22, 7.54)	7.38(7.23, 7.54)	0.98

[a] Crude linear mixed models without adjusting for covariates, core menu items

Table S11 Per-item Energy and Nutrient Changes by Food Category, Unadjusted Models, All Menu Items (N = 23,911)

Food Category	Predicted per-item change (95%CI) **Energy (kcal)** [a]	Predicted per-item change (95%CI) **Salt (g)** [a]	Predicted per-item change (95%CI) **Sugar (g)** [a]	Predicted per-item change (95%CI) **Saturated Fat (g)** [a]
Appetizers & sides **N = 2,316**	3.47(-11.70, 18.65)	**0.08(0.00, 0.16)** [b]	0.41(-0.29, 1.11)	0.04(-0.33, 0.40)
Baked goods **N = 1,178**	**34.00(11.24, 56.77)** [b]	0.07(-0.05, 0.19)	**1.56(0.51, 2.61)** [b]	**1.27(0.72, 1.82)** [b]
Beverages **N = 6, 490**	4.56(-5.23, 14.35)	0.02(-0.03, 0.07)	**-0.91(-1.36, -0.46)** [b]	**0.62(0.38, 0.86)** [b]
Burgers **N = 850**	-24.43(-49.69, 0.84)	0.00(-0.14, 0.13)	-0.14(-1.30, 1.02)	**-1.11(-1.72, -0.49)** [b]
Desserts **N = 1,536**	**-36.24(-54.73, -17.74)** [b]	-0.05(-0.15, 0.04)	**-2.94(-3.80, -2.09)** [b]	**-0.53(-0.98, -0.08)** [b]
Mains **N = 2,892**	**34.05(20.24, 47.85)** [b]	**0.21(0.14, 0.28)** [b]	0.18(-0.47, 0.83)	-0.04(-0.38, 0.31)
Fried potatoes **N = 383**	-29.86(-66.22, 6.49)	-0.02(-0.21, 0.18)	-0.87(-2.54, 0.81)	-0.85(-1.74, 0.04)
Pizza **N = 4,262**	**-19.98(-30.73, -9.23)** [b]	**-0.09(-0.15, -0.04)** [b]	-0.28(-0.78, 0.21)	-0.31(-0.57, -0.05)
Salads **N = 633**	-13.43(-43.16, 16.30)	-0.10(-0.25, 0.06)	0.08(-1.29, 1.45)	-0.38(-1.11, 0.34)
Sandwiches **N = 1,539**	**-21.90(-41.87, -1.92)** [b]	**-0.18(-0.28, -0.07)** [b]	-0.86(-1.78, 0.06)	-0.09(-0.57, 0.39)
Soups **N = 249**	1.23(-47.46, 49.91)	0.09(-0.17, 0.34)	-0.36(-2.60, 1.88)	-0.57(-1.75, 0.61)
Toppings & ingredients **N = 1,583**	13.81(-4.02, 31.63)	0.08(-0.01, 0.17)	-0.25(-1.07, 0.57)	0.27(-0.17, 0.70)

[a] Crude linear mixed models stratified by food group, all menu items
[b] Boldface indicates statistical significance ($p<0.05$)

Table S12 Per-item Energy and Nutrient Changes by Food Category, Unadjusted Models, Core Menu Items (N = 1,855)

**Food Category**	Predicted per-item change (95%CI) **Energy (kcal)** [a]	Predicted per-item change (95%CI) **Salt (g)** [a]	Predicted per-item change (95%CI) **Sugar (g)** [a]	Predicted per-item change (95%CI) **Saturated Fat (g)** [a]
Appetizers & sides **N = 182** [c]	0.09(-8.45, 8.63)	0.05(-0.01, 0.10)	-0.08(-0.52, 0.35)	0.07(-0.18, 0.32)
Baked goods **N = 105**	**18.04(6.87, 29.20)** [b]	0.06(-0.01, 0.14)	-0.04(-0.59, 0.51)	0.26(-0.05, 0.58)
Beverages **N = 265**	-1.26(-8.38, 5.87)	-0.01(-0.05, 0.04)	**-1.38(-1.73, -1.02)** [b]	-0.11(-0.31, 0.10)
Burgers **N = 67**	0.13(-13.80, 14.06)	-0.03(-0.12, 0.07)	0.03(-0.65, 0.72)	-0.15(-0.54, 0.24)
Desserts **N = 93**	-4.68(-16.56, 7.21)	-0.01(-0.09, 0.07)	-0.52(-1.11, 0.07)	-0.27(-0.61, 0.06)
Mains **N = 182**	**-11.39(-19.84, -2.94)** [b]	0.05(-0.01, 0.10)	0.18(-0.24, 0.60)	**-0.53(-0.77, -0.29)** [b]
Fried potatoes **N = 28**	-8.45(-30.29, 13.38)	0.00(-0.14, 0.14)	-0.38(-1.45, 0.69)	0.28(-0.33, 0.89)
Pizza **N = 709**	3.14(-1.20, 7.48)	-0.01(-0.04, 0.02)	-0.20(-0.41, 0.01)	**0.20(0.08, 0.32)** [b]
Salads **N = 35**	2.91(-16.36, 22.18)	-0.01(-0.14, 0.11)	-0.04(-1.00, 0.92)	-0.16(-0.71, 0.38)
Sandwiches **N = 64**	0.55(-13.79, 14.88)	-0.03(-0.12, 0.07)	-0.10(-0.80, 0.61)	**-0.44(-0.85, -0.04)** [b]
Soups **N = 15**	3.89(-26.30, 34.07)	-0.04(-0.24, 0.15)	0.04(-1.45, 1.52)	0.06(-0.79, 0.91)
Toppings & ingredients **N = 110**	0.78(-10.18, 11.73)	0.03(-0.04, 0.10)	-0.24(-0.78, 0.30)	0.04(-0.27, 0.35)

[a] Crude linear mixed models stratified by food group, all menu items
[b] Boldface indicates statistical significance ($p<0.05$)
[c] Number of core menu items, each core menu item has three data points (2018, 2019, 2020)
